# Crowdsourcing-based nationwide tick collection reveals the distribution of *Ixodes ricinus* and *I. persulcatus* and associated pathogens in Finland

**DOI:** 10.1038/emi.2017.17

**Published:** 2017-05-10

**Authors:** Maija Laaksonen, Eeva Sajanti, Jani J Sormunen, Ritva Penttinen, Jari Hänninen, Kai Ruohomäki, Ilari Sääksjärvi, Eero J Vesterinen, Ilppo Vuorinen, Jukka Hytönen, Tero Klemola

**Affiliations:** 1 Department of Biology, University of Turku, Turku FI-20014, Finland; 2 Department of Medical Microbiology and Immunology, University of Turku, Turku FI-20520, Finland; 3 Archipelago Research Institute, Biodiversity Unit, University of Turku, Turku FI-20014, Finland; 4 Zoological Museum, Biodiversity Unit, University of Turku, Turku FI-20014, Finland; 5 Department of Agricultural Sciences, University of Helsinki, Helsinki FI-00014, Finland

**Keywords:** *Borrelia burgdorferi*, *Borrelia miyamotoi*, crowdsourcing, distribution, *Ixodes persulcatus*, *Ixodes ricinus*, tick-borne encephalitis virus, tick-borne pathogens

## Abstract

A national crowdsourcing-based tick collection campaign was organized in 2015 with the objective of producing novel data on tick distribution and tick-borne pathogens in Finland. Nearly 20 000 *Ixodes* ticks were collected. The collected material revealed the nationwide distribution of *I. persulcatus* for the first time and a shift northwards in the distribution of *I. ricinus* in Finland. A subset of 2038 tick samples containing both species was screened for *Borrelia burgdorferi* sensu lato (the prevalence was 14.2% for *I. ricinus* and 19.8% for *I. persulcatus*), *B. miyamotoi* (0.2% and 0.4%, respectively) and tick-borne encephalitis virus (TBEV; 0.2% and 3.0%, respectively). We also report new risk areas for TBEV in Finland and, for the first time, the presence of *B. miyamotoi* in ticks from mainland Finland. Most importantly, our study demonstrates the overwhelming power of citizen science in accomplishing a collection effort that would have been impossible with the scientific community alone.

## INTRODUCTION

Ticks are the primary vectors for several zoonotic infections worldwide. Ticks and tick-transmitted pathogens are presently under active investigation due to the status of tick-borne diseases as emerging infections. The most important tick-borne diseases in Finland are Lyme borreliosis (LB; with ~1900 microbiologically confirmed cases yearly and estimated 6000–7000 total cases yearly; ~120 cases per 100 000 individuals) and tick-borne encephalitis (TBE; with ~40–60 microbiologically confirmed cases yearly; ~1 case per 100 000 individuals) according to the National Infectious Disease Register maintained by National Institute for Health and Welfare (https://www.thl.fi/ttr/gen/rpt/tilastot.html). *Borrelia miyamotoi* is a spirochete belonging to the relapsing fever group of *Borrelia* with an unknown prevalence and geographic distribution in Finland. The distribution of tick species in Finland is exceptional because it is the northernmost border of tick distribution in Europe, and the distribution borders of two important tick species (*Ixodes ricinus* and *I. persulcatus)* are both located within the country. The distribution of these tick species and the diversity of their associated pathogens have never been intensely studied in Finland. Surveys conducted in neighboring countries suggest a northward shift in the distribution of *Ixodes* ticks as well as an increase in abundance over the past few decades.^1, 2, 3, 4^ However, the current tick situation in Finland and elsewhere in northern Europe has not been fully characterized.

Tick collection using the traditional methods such as cloth dragging and flagging is both time-consuming and laborious, and covers a relatively small geographical area in a certain time in most research frames. Large-scale sample collection cannot be carried out with a limited number of researchers. Crowdsourcing is utilized relatively rarely but is an effective method for gathering data in health-related research.^5^ To construct a comprehensive, nationwide collection of ticks, we launched a national campaign using an innovative crowdsourcing approach in which citizens were asked to participate in tick collection. The national tick collection campaign was organized in 2015, advertised on the internet, television and newspapers, and was a success. Approximately 7000 shipments were received containing nearly 20 000 individual ticks from all over Finland. The samples gathered formed the so-called ‘Tickbank’ and constitute unique material for ecological, taxonomical, medical and veterinary medical studies. Here we present the first results from this vast material.

## MATERIALS AND METHODS

### Tick collection and metadata gathering

From April to November 2015, citizens were asked to send ticks (dead or alive) via postal mail to the Department of Biology at the University of Turku as a part of the tick collection campaign. Along with the ticks, they were asked to provide information on the collection site and date, and the species of the possible host. This collection resulted in a Tickbank of 19 923 individual ticks. Ticks lacking adequate date information or collected outside the campaign period (*n*=1788) were stored in the Tickbank but were not used in the further analyses. The species, life stage and sex of tick samples were identified based on morphological characteristics under a microscope, if possible. Almost all the received samples were recognized correctly as *Ixodes* ticks by citizens; those that represented other species (for example, deer keds, spiders and moss mites) were not stored in the Tickbank or analyzed in this study. After identification, ticks were stored at −80 °C. The geographical information of the ticks was stored as ETRS-TM35FIN coordinates with an accuracy of 100 m. In most cases, the collection site information provided by citizens was accurate enough. In a minority of the cases (∼300), the collection site information was inaccurate and therefore those tick samples were not used in the distribution analyses. Distribution maps were created using MapInfo Professional 12.0 software (Pitney Bowes Business Insight, Troy, NY, USA).

### DNA and RNA extraction

A subset of 2038 ticks (1044 *I. ricinus* and 994 *I. persulcatus*) were selected for screening for *B. burgdorferi* s.l., *B. miyamotoi* and tick-borne encephalitis virus (TBEV). The samples were manually selected to represent the major collection areas, tick life stages and sex distribution of the whole Tickbank. However, we selected approximately the same number of *I. ricinus* and *I. persulcatus* samples to obtain a comprehensive picture of both species. DNA and RNA were extracted from the tick samples sequentially using NucleoSpin RNA kits and RNA/DNA buffer sets (Macherey-Nagel, Düren, Germany) following the kit protocols (RNA Kit: Rev. 16 May 2014 and RNA/DNA buffer set: Rev. 08 May 2014). RNA extracts were stored at −80 °C and DNA extracts were stored at −20 °C.

### Real-time PCR assays

Tick species, if unknown after morphological identification (*n*=98), was determined in a species-specific duplex real-time PCR assay as previously described^6^ (detailed protocol in Supplementary Materials). IXO-I2-F4 and IXO-I2-R4 primers targeting a 94-bp fragment of *Ixodes* spp. internal transcribed spacer 2 (*ITS2*) gene were used to amplify genus-specific segments, and Ipe-I2-P4 and Iri-I2-P4 probes were used to match the *ITS2* region for either tick species (*I. persulcatus* or *I. ricinus*, respectively; Table 1).^7, 8, 9, 10^ DNA samples from *I. ricinus* and *I. persulcatus* confirmed by sequencing in an earlier study^9^ were used as positive controls, and double-distilled water (ddH_2_O) was used as a negative control in each assay.

Bbsl-ospA-F and Bbsl-ospA-R primers, and a Bbsl-ospA-P probe (Table 1) amplifying a 102-bp fragment of the outer surface protein A (*ospA*) gene as previously described ^7^ were used to detect *B. burgdorferi* s.l. DNA (Supplementary Methods). Positive and negative controls (*B. burgdorferi* sensu stricto strain B31 ATCC 35210 and ddH_2_O, respectively) were included in all runs. For *B. miyamotoi*, Bm-fla-F and Bm-fla-R primers, and a Bm-fla-P probe (Table 1) targeting the *B. miyamotoi flagellin* gene (156 bp) were used as previously described^8^ with minor modifications. DNA samples from *B. miyamotoi* confirmed by sequencing in earlier studies^6, 11^ were used as positive controls; *B. burgdorferi* sensu stricto strain B31 (ATCC 35210) and ddH_2_O were used as negative controls.

For TBEV screening, aliquots of the original RNA samples were first pooled (10 samples per pool, 5 μL of each sample) because a low prevalence was expected. Then, the pools were examined using real-time reverse transcription-PCR with F-TBEV1 and R-TBEV1 primers, and a P-TBEV-WT probe (Table 1) amplifying the 3′-non-coding region of the TBEV genome as previously described^10, 12^ (Supplementary Materials). Individual RNA samples were re-analyzed if a pooled sample tested positive. Positive (TBEV-Sib and TBEV-Eur)^13, 14^ and negative (ddH_2_O) controls were included in each run.

### Statistical analyses

Data were managed using Microsoft Excel 2013 (Redmond, WA, USA). Because the independence of observations is an underlying assumption of most basic statistical tests, statistical analysis of citizen-collected data is a challenging task. On many occasions, we received many ticks in one letter, indicating that these ticks were dependent on each other, for example, similar collection times, locations, hosts, and often by tick species and developmental stage. Therefore, we refrained from formal statistical analyses apart from testing one specific hypothesis and controlling for dependent observations (see below).

**Figure 1:**
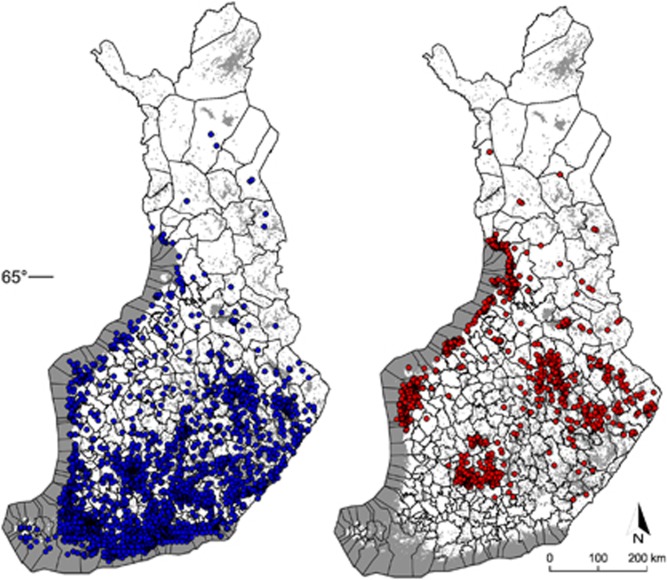
Map illustrating the distribution of *I. ricinus* and *I. persulcatus* in Finland based on the coordinates of 17 603 tick samples collected in 2015 via the collection campaign. Blue dots indicate collection points for *I. ricinus* (*n*=13  847) and red dots indicate collection points for *I. persulcatus* (*n*=3756).

Previous studies have suggested a higher prevalence of *B. burgdorferi* s.l. among samples of *I. persulcatus* compared to *I. ricinus*.^15, 16, 17^ We tested this hypothesis using a generalized linear mixed model (GLMM) for the screened adults of both tick species. Larvae and nymphs were ignored because of their low sample sizes (Supplementary Table S1). To separate the possible effect of tick species from that of dissimilar environments (for example, due to weather or distance to the southern coast; Figure 1), we restricted the analysis to *I. persulcatus* (*n*=885; 658 females and 227 males) and *I. ricinus* (*n*=527; 393 females and 134 males) samples collected from the area of their sympatric occurrence. In practice, this was done by simply filtering the data according to the N coordinate of the southernmost *I. persulcatus* and northernmost *I. ricinus*.

We modeled the probability of an adult tick testing positive for *B. burgdorferi* s.l. by running a generalized estimating equation, a specific type of GLMM for clustered observations, with a binomial error distribution and logit link function. The shipment ID was set as a clustering factor, whereas the species and sex of the tick were fixed explanatory factors. The model was run with the GENMOD procedure in SAS statistical software, v. 9.4. (Cary, NC, USA).^18^

## RESULTS

### Characteristics of the Tickbank

Our crowdsourcing-based tick collection was extremely successful, with nearly 7000 shipments from all over Finland. These resulted in the Tickbank of 19 923 individual ticks, of which nearly 80% were *I. ricinus*. After samples lacking adequate date or collection site information were excluded, the remaining 17 603 coordinates were used in distribution analyses. Most tick samples were received from central Finland (the so-called Finnish Lakeland) and coastal areas (Figure 1). A considerable number of ticks were received from the southern coast of Finland, all of which were *I. ricinus*. The northernmost collection sites were beyond the Arctic Circle, at latitudes of 67° N in Lapland. Both tick species were received from northern Finland (north of latitude 65° N), although almost all of these samples (97% 760/784) were *I. persulcatus.* Whereas *I. ricinus* was more evenly distributed over southern and eastern Finland, and the coastal areas, *I. persulcatus* seemed to have three distinct clusters in distribution: on the coast of the Gulf of Bothnia, in eastern Finland and in the middle of southern Finland (Figure 1).

Of the ticks collected in 2015 (*n*=18 135), 17 936 could be identified morphologically to species (14 133 *I. ricinus* and 3803 *I. persulcatus*). Of these, most were adults (Table 2). *I. ricinus* samples contained relatively more young developmental stages (larvae and nymphs) than *I. persulcatus* (5.5% vs. 1.1%, respectively). Adult samples were more often female (*n*=12 246) than male (*n*=4880), with similar proportions for both species. The most frequently reported host was dog (54.2% for *I. ricinus* and 62.2% for *I. persulcatus*). *I. persulcatus* was detected more often in humans (19.7% vs. 14.5%) whereas *I. ricinus* was found more often in cats (30.3% vs. 17.3% Table 2).

**Figure 2:**
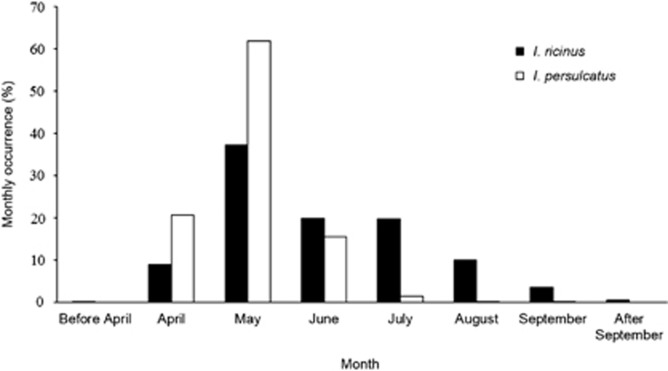
A diagram showing the monthly occurrence of *I. ricinus* and *I. persulcatus* samples collected via the collection campaign.

Most of the ticks were collected in May (Figure 2). *I. persulcatus* was collected mainly from April to June (98.1%), whereas the collection period for *I. ricinus* was more evenly distributed throughout the summer and early autumn. *I. persulcatus* was apparently no longer active in October and November, whereas almost 100 *I. ricinus* individuals were collected during the same period.

### The subset of 2038 ticks—characteristics and pathogen screening results

**Figure 3:**
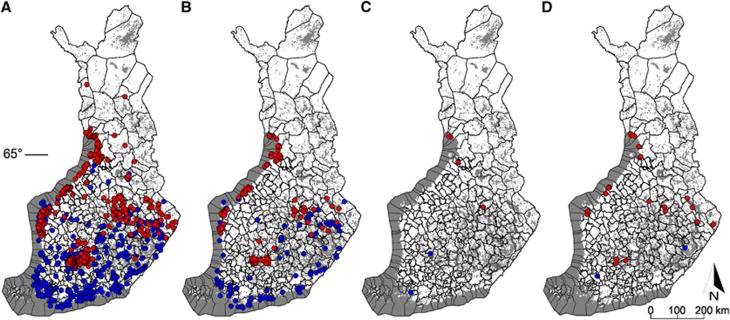
(**A**) Distribution of the samples that were screened for pathogens (*n*=2038). Blue dots indicate collection points for *I. ricinus* samples (*n*=1044) and red dots indicate collection points for *I. persulcatus* samples (*n*=994). (**B**) Distribution of the samples that were positive for *Borrelia burgdorferi* s.l. (*n*=345). (**C**) Distribution of the samples that were positive for *B. miyamotoi* (*n*=6). (**D**) Distribution of the samples that were positive for TBEV (*n*=32).

A total of 1044 *I. ricinus* and 994 *I. persulcatus* were selected for screening of *B. burgdorferi* s.l., *B. miyamotoi* and TBEV. These ticks represented the whole Tickbank in terms of collection site (Figures 1 and 3A), tick life stage and sex distribution, and reported host (Table 2; Supplementary Table S1). Of ticks that could not be identified morphologically by microscope, 57 were identified as *I. ricinus* and 41 as *I. persulcatus* by duplex real-time PCR.

In total, *B. burgdorferi* s.l. was detected in 16.9% (345/2038) of the screened DNA samples (Table 3). The prevalence was 14.2% (148/1044) for *I. ricinus* and 19.8% (197/994) for *I. persulcatus*. Divided by stages, the prevalence of *B. burgdorferi* s.l. was 17.1% (332/1945) for adult ticks and 14.4% (13/91) for nymphs. No larvae were found to be infected. The GLMM conducted for the adults in the sympatric region indicated a significantly higher probability of a positive finding for *I. persulcatus* (the estimated marginal mean (with 95% confidence interval) was 0.196 (0.166–0.232)) compared with that of *I. ricinus* (0.137 (0.106–0.174); Wald statistics, species: *χ*^2^=5.67, DF=1, *P*=0.017). No differences in the prevalence of *B. burgdorferi* s.l. were observed between females and males of either species (sex: *χ*^2^=1.03, DF=1, *P*=0.311; species × sex: *χ*^2^=0.03, DF=1, *P*=0.872). The distribution map drawn from the positive *B. burgdorferi* s.l. samples corresponded to the distribution of the whole subset of ticks (Figure 3B). *B. miyamotoi* was detected in six DNA samples, of which two were *I. ricinus* (0.2% 2/1044) and four *I. persulcatus* (0.4% 4/994). All of the *B. miyamotoi*-positive ticks were adults collected from southwestern Finland, central Finland and the coast of the Bothnian Bay (Figure 3C). Two ticks, both *I. persulcatus*, were co-infected with *B. burgdorferi* s.l. and *B. miyamotoi*.

Of 2038 screened RNA samples, 32 (1.6%) were TBEV positive (Table 3). The prevalence of TBEV was higher for *I. persulcatus* (3.0% 30/994) than for *I. ricinus* (0.2% 2/1044). One of the positive *I. persulcatus* samples was a nymph, but all others were adult ticks. TBEV-positive samples were collected from coastal areas in the Bothnian Bay, eastern Finland and south-central Finland (Figure 3D). Eight ticks (two males and six females), all *I. persulcatus*, were co-infected with TBEV and *B. burgdorferi* s.l.

## DISCUSSION

Crowdsourcing is utilized relatively infrequently to solve scientific issues and gather data in health-related research.^5^ Using this novel method of collecting citizen-contributed samples, we succeeded in constructing a large and geographically comprehensive collection of ticks, the Tickbank. Using the collected material, we investigated the distribution of two tick species, *I. ricinus* and *I. persulcatus*, and the prevalence of tick-associated pathogens in Finland. Compared with the previous nationwide distribution map drawn according to a survey in Finland almost 60 years ago,^19^ the extent of spatial distribution for ticks has shifted 200–300 km northwards and populations have become established in new locations, mainly in coastal areas of the Bothnian Bay and in the eastern part of central Finland. Most of the ticks received were from the coastlines and around Finnish Lakeland, perhaps because of the dry continental climate elsewhere that is suboptimal for ticks. The northernmost tick samples were from latitudes of 67° N. However, only a few ticks were received from this latitude, thus one may speculate whether they came from stable populations or may be stragglers that were transported there by migratory birds, cervids or pet animals.

The observed extension in tick distribution in our study is in accordance with other studies conducted in Europe. Climate change is thought to be a major factor driving changes in tick distribution and abundance, through milder winters and extended growing seasons in the northern hemisphere, faster tick developmental rates and changes in the abundance of host animals.^2, 3, 20, 21, 22, 23, 24, 25, 26^ In Finland, the increase in the temperature has been remarkably rapid since the late 1960s,^27^ and at the same time, the ticks’ host animals have become more abundant.^28, 29, 30, 31, 32, 33^ However, this study and the survey conducted in 1956–1958^19^ are not entirely comparable, due to different extents and methods used to determine the tick distribution (unselected vs. selected sampling).

The majority of received ticks were *I. ricinus* collected from urbanized areas in southern Finland, likely due to a higher human population density. However, *I. persulcatus* is now also widely established in Finland and is even more abundant than *I. ricinus* in certain areas. For instance, in northern Finland, *I. persulcatus* is clearly the dominant tick species. Previous studies suggest that *I. persulcatus* is more cold-resistant than *I. ricinus*,^34^ and hence could potentially survive better in the north. In contrast, all the samples from the southern coast of Finland were *I. ricinus*. However, *I. persulcatus* can be found in corresponding latitudes in Russian Karelia^35^ and even further south in Estonia and Latvia.^36^ This observation may be related to tick reproduction. In principle, *I. ricinus* and *I. persulcatus* can interbreed, but the offspring are sterile.^37^ Thus, it may be difficult for one species to gain ground in a new area where the other species is already established. This could partly explain why there are no established populations of *I. persulcatus* in the southern coast of Finland, where *I. ricinus* has long been abundant. In addition, possible species-specific landscape and biotopic preferences, different seasonal activity patterns, and other biological characteristics may have an influence. The exact reasons for the dominance of *I. ricinus*, and lack of *I. persulcatus*, in southern Finland remain unknown.

Over twice as many females as males were collected, with similar proportions for both species. Most of the collected samples were adults, probably due to the better visibility of adults and longer questing periods of adult females compared to nymphs and larvae. *I. ricinus* samples contained relatively more young developmental stages (nymphs and larvae) than *I. persulcatus* samples. According to previous observations, *I. ricinus* commonly attaches to people at the nymphal stage, whereas *I. persulcatus* prefers to do so at the adult stage.^15, 38^ Furthermore, in our study, *I. persulcatus* was collected from humans five percentage points more often. However, the most commonly reported host for both tick species was dog. *I. persulcatus* was detected from dogs eight percentage points more often, whereas *I. ricinus* was detected from cats over ten percentage points more often. This observation may be related to the different outdoor activity habits of cats compared to dogs and humans. However, samples collected from the reported individual host could include largely varying numbers of ticks, both attached and unattached, which could cause a bias in the frequencies of the reported host animals. Further studies of possible differences related to host animal preferences of *I. ricinus* and *I. persulcatus* are needed.

*I. ricinus* were collected throughout the summer months, whereas *I. persulcatus* were collected mostly during early summer, especially in May. Previous studies have shown that the seasonal activity of *I. ricinus* adults and nymphs is mainly two-peaked, whereas *I. persulcatus* adults have only one spring activity peak and are found to be questing only until July.^34, 39^

The subset of 2038 ticks selected for the pathogen screening represented the whole tick collection in terms of collection site, sex and developmental stage distribution, and reported hosts. However, due to our sampling method, a higher proportion of *I. ricinus* samples collected in May and June was analyzed for pathogens (84.1%) compared to their proportion in the whole Tickbank (60.5%).

Of the 1044 analyzed *I. ricinus* and 994 *I. persulcatus* ticks, 148 (14.2%) and 197 (19.8%) were positive for *B. burgdorferi* s.l., respectively. The results of the previous studies of *Borrelia* prevalence conducted in Europe vary among years and according to the methods used. In a meta-analysis from Europe, the prevalence of *B burgdorferi* s.l. in *I. ricinus* adults was 18.6%.^40^ Furthermore, there are great variations in tick infection rates within the country: in a study conducted in southwestern Finland in 2015, *B. burgdorferi* s.l. was detected in 23.5% of adult *I. ricinus* ticks,^6^ but a prevalence of up to 55% was reported in a study conducted in recreational parks in Helsinki in 1999.^41^ When investigating the prevalence of *B. burgdorferi* s.l. in the sympatric region only (excluding samples from the north and the southern coast of Finland), a lower prevalence was still observed for *I. ricinus* than for *I. persulcatus* adults. Shipment ID as a clustering factor was also found to influence prevalence, meaning that positive samples were correlated with the same sender. Our finding of a higher prevalence of *B. burgdorferi* s.l. in *I. persulcatus* than in *I. ricinus* ticks has also been observed in previous studies conducted in sympatric regions.^15, 16, 17^ As expected,^40^ the prevalence of *B. burgdorferi* s.l. appears to be higher in adults (17.1%) than in nymphs (14.3%) in the current study. This is the first report of *B. miyamotoi* in ticks from mainland Finland. *B. miyamotoi* was found in 6 out of 2038 (0.3%) ticks, which is approximately in accordance with the results of studies conducted in neighboring countries.^11, 42, 43^

The overall prevalence of TBEV was 1.6%. A TBEV prevalence of 0.2–2.0% has been reported in questing ticks in TBE-endemic areas in Europe.^44^ However, the annual prevalence of TBEV in ticks even in one site can vary remarkably.^39^ As the transmission cycle of TBEV is fragile, microclimatic conditions may affect its survival in nature^45^ and thus TBEV might not be distributed equally. This was observed also in our study, with positive samples aggregating in clear clusters. Moreover, some of the positive samples were correlated to the same collectors and collection sites. All but one of the TBEV-positive ticks were adults, probably due to the relatively small number of nymphs. As in the case of *B. burgdorferi* s.l., a higher TBEV prevalence was observed in *I. persulcatus* (1.6%) than in *I. ricinus* (0.2%), which has also been found in previous studies.^39^

In addition to previously known tick endemic areas, we received TBEV-positive ticks from areas where only sporadic TBE cases have been reported. Four samples were obtained from the Tampere region in the middle of southern Finland, suggesting a new TBEV endemic focus. This new focus area is inhabited by over 350 000 citizens and thus could pose an emerging threat to the local human population. Interestingly, none of the ticks from the southern and southwestern coasts of Finland were positive for TBEV, where the majority of human TBE cases in Finland are reported. This may be related to the clustered nature of TBEV distribution, as well as the low expected prevalence of the virus in nature; in other words, coincidence.

Although crowdsourcing is an effective method for gathering data, it inevitably has some limitations that can affect the generalization and reliability of the results. Because the samples are gathered by citizens instead of professional scientists, we cannot be sure of the reliability of all the collection information, such as the exact collection site or date. Moreover, it is possible that volunteer citizens are especially interested in ticks, which could cause a bias in the amount of ticks collected in certain areas. However, with nearly 7000 shipments received from all over the country, we expect that not only those especially interested in ticks participated, even when some of the shipments were received from the same sender. For ecological research, the collection is definitely biased by the proportion of different tick life stages. It is also obvious that most of the samples were received from the highly populated areas of Finland. However, a substantial proportion of the tick samples was also received from the sparsely inhabited areas, such as eastern Finland. Thus, we can conclude that the areas from where we received *B. burgdorferi* s.l.-infected ticks are obvious risk areas to obtain LB, but we cannot exclude the possibility that there are infected ticks in those areas that appear ‘white’ on our map. Interestingly, the map drawn from the incidence of microbiologically confirmed LB infections in Finland in 2015^46^ substantially overlaps our map of tick distribution, further strengthening the idea that the main tick distribution areas presented in this study are indeed the areas of high risk for LB.

In the present study, we report the first results from this unique national tick collection. Ten percent of the tick samples in the Tickbank were analyzed in this study, and this subset of 2038 samples gives us a reliable overview of *B. burgdorferi* s.l. and TBEV prevalence in Finnish ticks. Ongoing global climate change is expected to cause more changes in tick abundance and distribution patterns in future years, along with changes in tick-borne pathogen diversity and prevalence. To investigate temporal changes in tick distribution and pathogen diversity, a new collection of tick samples will be necessary in the future. However, for now, the Tickbank offers an exceptionally comprehensive overview of ticks and tick-borne pathogens in Finland. Finally, our tick assemblage offers a significant perspective on the emergence of rare and new potentially dangerous pathogens that would go undetected in a smaller collection effort.

## Figures and Tables

**Table 1:** Primers and probes used in tick species determination and pathogen screening

**Primer/probe name**	**Target name**	**Nucleotide sequence (5**′**→3**′**)**	**Reference**
*Real-time PCR*			
Bbsl-ospA-F	*B. burgdorferi ospA*	AATATTTATTGGGAATAGGTCTAA	7
Bbsl-ospA-R		CACCAGGCAGCAAATCTACTGA	
Bbsl-ospA-P		[6FAM]-TTAATAGCATGTAAGCAAAATGTTAGCA-[DDQ1]	
Bm-fla-F	*B. miyamotoi flagellin*	AGAAGGTGCTCAAGCAG	8
Bm-fla-R		TCGATCTTTGAAAGTGACATAT	
Bm-fla-P		[6FAM]-AGCACAACAGGAGGGAGTTCAAGC-[DDQ1]	
IXO-I2-F4	*Ixodes* spp. *ITS2*	TCTCGTGGCGTTGATTTGC	9
IXO-I2-R4	*Ixodes* spp. *ITS3*	CTGACGGAAGGCTACGACG	
Ipe-I2-P4	*I. persulcatus ITS4*	[FAM]-TGCGTGGAAAGAAAACGAG-[BHQ1]	
Iri-I2-P4	*I. ricinus ITS5*	[VIC]-TGCTCGAAGGAGAGAACGA-[BHQ1]	

*Real-time RT-PCR*			
F-TBEV1	3′-non-coding region of the TBEV genome	GGGCGGTTCTTGTTCTCC	10
R-TBEV1		ACACATCACCTCCTTGTCAGACT	
P-TBEV-WT		[FAM]-TGAGCCACCATCACCCAGACACA-[TAMRA]	

Abbreviations: internal transcribed spacer 2, ITS2; outer surface protein A, ospA; reverse transcription-PCR, RT-PCR; tick-borne encephalitis virus, TBEV.

**Table 2:** Information for the samples collected in 2015 via the collection campaign

	**Number (%) of** ***I. ricinus*** **samples**	**Number (%) of** ***I. persulcatus*** **samples**	**Total**
Amount	14 133 (78.8)	3803 (21.2)	17 936 (100.0)

*Sex of adult ticks*			
Female	9555 (71.5)	2691 (71.6)	12 246 (71.5)
Male	3810 (28.5)	1070 (28.4)	4880 (28.5)
Total	13 365 (100.0)	3761 (100.0)	17 126 (100.0)

*Developmental stage*			
Adult	13 365 (94.5)	3761 (98.9)	17 126 (95.5)
Nymph	743 (5.3)	41 (1.1)	784 (4.4)
Larva	25 (0.2)	1 (0.0)	26 (0.1)
Total	14 133 (100.0)	3803 (100.0)	17 936 (100.0)

*Collected from*			
Dog	7289 (54.2)	2195 (62.2)	9484 (55.9)
Cat	4075 (30.3)	609 (17.3)	4684 (27.6)
Human	1945 (14.5)	695 (19.7)	2640 (15.6)
Other animal	88 (0.7)	2 (0.0)	90 (0.5)
Nature	46 (0.3)	27 (0.8)	73 (0.4)
Total	13 443 (100.0)	3528 (100.0)	16 971 (100.0)

*Of all these samples (*n*=18 135), 17 936 were identified as *Ixodes ricinus* or *I. persulcatus*; 199 samples could not be identified.

Each category (sex, developmental stage and collected from) contains missing data, such that the total amount differs from the total number of collected ticks. ‘Other animal’ (*n*=90) includes animals such as horse, sheep, raccoon dog, European roe deer and white-tailed deer.

**Table 3:** Prevalence (%) of the studied pathogens in *I. ricinus* and *I. persulcatus* samples

	**Number (%) of samples positive for** ***B. burgdorferi*** **s.l.**			**Number (%) of samples positive for** ***B. miyamotoi***			**Number (%) of samples positive for TBEV**		
	**R**	**P**	**Total**	**R**	**P**	**Total**	**R**	**P**	**Total**
Total	148 (14.2)	197 (19.8)	345 (16.9)	2 (0.2)	4 (0.4)	6 (0.3)	2 (0.2)	30 (3.0)	32 (1.6)

*Sex*									
Female	99 (13.1)	138 (19.0)	237 (16.0)	2 (0.3)	1 (0.1)	3 (0.2)	2 (0.3)	23 (2.3)	25 (1.7)
Male	37 (17.3)	57 (22.9)	94 (20.3)	0	3 (1.2)	3 (0.6)	0	7 (2.8)	7 (1.5)

*Stage*									
Adult	137 (14.1)	195 (20.0)	332 (17.1)	2 (0.2)	4 (0.4)	6 (0.3)	2 (0.2)	29 (3.0)	32 (1.6)
Nymph	11 (15.1)	2 (11.8)	13 (14.3)	0	0	0	0	1 (5.6)	1 (1.1)
Larva	0	0	0	0	0	0	0	0	0

Abbreviations: *I. persulcatus*, P; *I. ricinus*, R; tick-borne encephalitis virus, TBEV.
