# Supplementary material for: Crowdsourcing-based nationwide tick collection reveals the distribution of Ixodes ricinus and I. persulcatus and associated pathogens in Finland
Source: Emerg Microbes Infect. 2017 May 10;6(5):e31–. doi: 10.1038/emi.2017.17 (PMC5584484; doi:10.1038/emi.2017.17)
Supplement: Supplementary Information [file emi201717x1.docx]

**Supplementary Materials**

**Methods**

Real-time PCR for tick species determination

Assays were carried out in 5μl reaction volume including 2.5μl KAPAProbe FAST PCR Kit (KAPA Biosystems, Wilmington, MA, USA), 200nM forward and reverse primers, 150nM Iri-I2-P4 probe (VIC), 100nM Ipe-I2-P4 probe (FAM), 1.175μl of ddH_2_O, and 1μl of template DNA. The thermal cycling conditions were: 95°C for 3min, 50 cycles of 95°C for 3s and 60°C for 30s. Real-time PCR (rPCR) runs were performed at the Finnish Microarray and Sequencing Centre (FMSC, Turku, Finland) using QuantStudio 12 K Flex Real-Time PCR System (Life Technologies Inc. [LTI], Carlsbad, CA, USA).

Real-time PCR and rRT-PCR for pathogen screening

The rPCR amplification of *ospA* gene (*B. burgdorferi* s.l.) was carried out in a final reaction volume of 20µl containing 600nM primers (Bbsl-ospA-F and Bbsl-ospA-R) and 100 nM probe (Bbsl-ospA-P), 10µl of LightCycler 480 Probes Master (Roche Diagnostics GmbH, Mannheim, Germany), 5µl of ddH_2_O, and 2µl of template DNA. The thermal cycling was performed under the following conditions: 95°C for 10min, 55 cycles of 95°C for 10s and 58°C for 1 min, followed by cooling at 40°C for 1min.

For *flagellin* gene rPCR (*B. miyamotoi*), the final reaction volume of 20µl was used including 200nM forward and reverse primers (Bm-fla-F and Bm-fla-R) and probe (Bm-fla-P) each, 10µl of LightCycler 480 Probes Master (Roche), 2.6µl of ddH_2_O, and 5µl of template DNA. The thermal cycling conditions were: 95°C for 5min, 60 cycles of 95°C for 5s and 60°C for 35s, cooling at 37°C for 1min. Real-time PCR runs for detecting *B. burgdorferi* s.l. and *B. miyamotoi* were carried out by LightCycler 480 II Real-Time PCR Instrument by Roche.

TBEV RNA was detected by one-step rRT-PCR in a final reaction volume of 5µl including 200nM forward primer F-TBEV1, 200nM reverse primer R-TBEV1, and 100nM probe P-TBEV-WT, 2.5µl 2x SensiFAST Probe Lo-ROX OneStep Mix (Bioline, London, UK), and 1.9µl template RNA. The rRT-PCR was run with the following protocol: 48⁰C for 10min (reverse transcription), followed by 95°C for 2min (polymerase activation) prior to the amplification of 50 cycles of 95°C for 5s (denaturation) and 60°C for 30s (annealing/extension). The TBEV assays were carried out at FMSC using QuantStudio 12 K Flex Real-Time PCR System (LTI).
