# Supplementary material for: Crowdsourcing-based nationwide tick collection reveals the distribution of Ixodes ricinus and I. persulcatus and associated pathogens in Finland
Source: Emerg Microbes Infect. 2017 May 10;6(5):e31–. doi: 10.1038/emi.2017.17 (PMC5584484; doi:10.1038/emi.2017.17)
Supplement: Supplementary Table S1 [file emi201717x2.docx]

**Supplementary Table S1**

**Results**

**Table 1.** Characteristics of the subset of 2,038 ticks selected for pathogen screening.

|  | No. (%) of *I. ricinus* samples | | No. (%) of *I. persulcatus*  samples | | Total |
| --- | --- | --- | --- | --- | --- |
| Amount | 1044 (51.2) | | 994 (48.8) | | 2038 (100.0) |
| Sex of adult ticks: |  | |  | |  |
| Female | 753 (77.7) | | 727 (74.5) | | 1480 (76.1) |
| Male | 216 (22.3) | | 249 (25.5) | | 463 (23.9) |
| Total | 969 (100.0) | | 976 (100.0) | | 1945 (100.0) |
| Developmental stage: | |  | |  | |
| Adult | 969 (92.8) | | 976 (98.2) | | 1945 (95.4) |
| Nymph | 73 (7.0) | | 18 (1.8) | | 91 (4.5) |
| Larva | 2 (0.2) | | 0 (0.0) | | 2 (0.1) |
| Total | 1044 (100.0) | | 994 (100.0) | | 2038 (100.0) |
| Collected from: |  | |  | |  |
| Dog | 500 (52.9) | | 579 (61.7) | | 1079 (57.3) |
| Cat | 329 (34.8) | | 146 (15.6) | | 475 (25.2) |
| Human | 107 (11.3) | | 208 (22.2) | | 315 (16.7) |
| Other animal | 7 (0.7) | | 2 (0.2) | | 9 (0.5) |
| Nature | 3 (0.3) | | 3 (0.3) | | 6 (0.3) |
| Total | 946 (100.0) | | 938 (100.0) | | 1884 (100.0) |
